# Supplementary material for: The Construction and Exploration of a Comprehensive MicroRNA Centered Regulatory Network in Foxtail Millet (Setaria italica L.)
Source: Front Plant Sci. 2022 May 6;13:848474. doi: 10.3389/fpls.2022.848474 (PMC9121102; doi:10.3389/fpls.2022.848474)
Supplement: Supplementary file 4 [file Data_Sheet_4.zip › Sit-miR2275b_Seita.3G297700.1_274_TPlot.pdf]

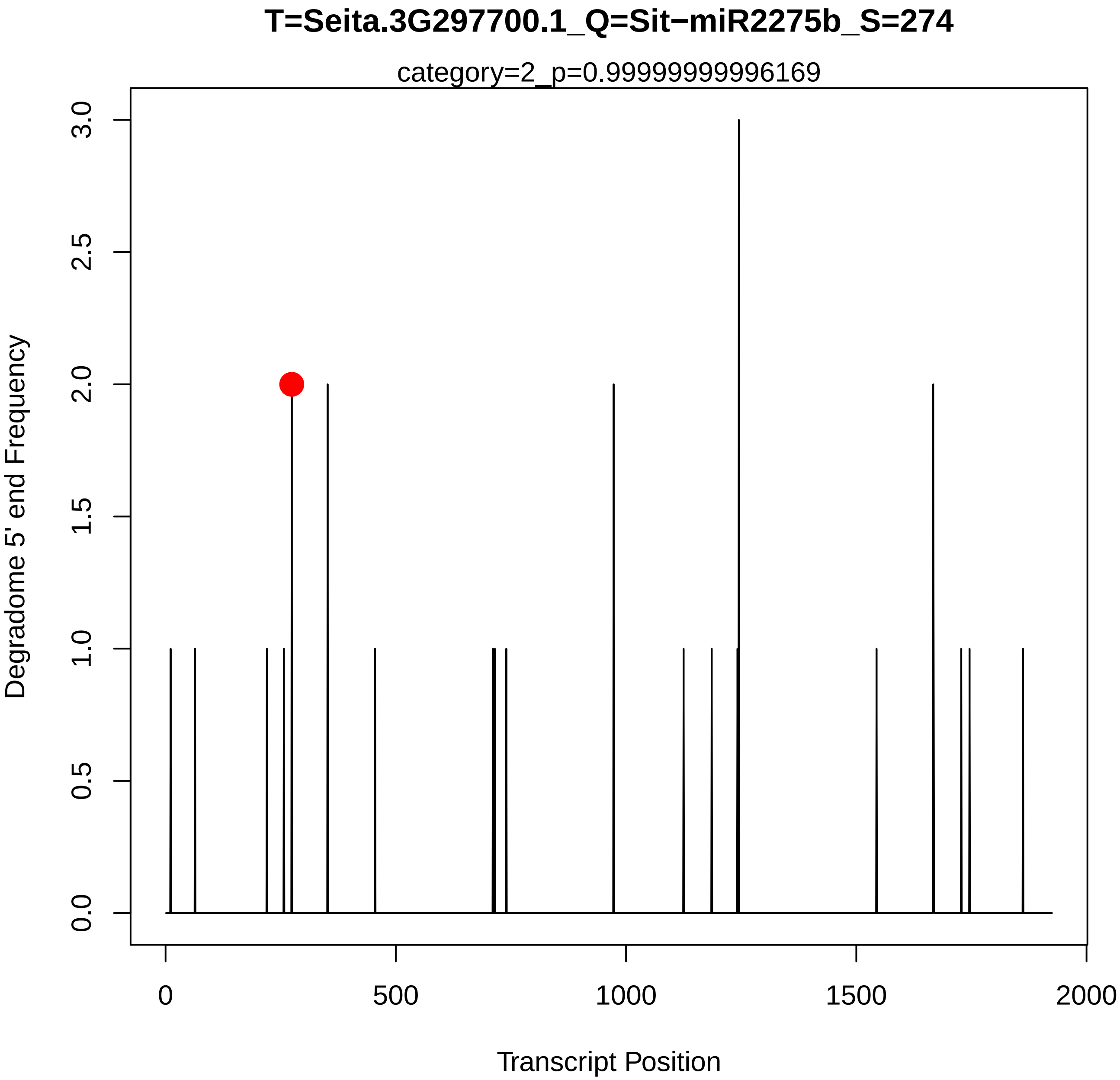
T=Seita.3G297700.1_Q=Sit−miR2275b_S=274
category=2_p=0.99999999996169
Degradome 5' end Frequency
0.0
0.5
1.0
1.5
2.0
2.5
3.0
0
500
1000
1500
2000
Transcript Position
